# COVID-19 associated Mucormycosis among ICU patients: risk factors, control, and challenges

**DOI:** 10.1186/s13568-023-01599-8

**Published:** 2023-09-22

**Authors:** Rehab Mahmoud Abd El-Baky, Esraa R. Shady, Ramadan Yahia, Fatma Y. Ahmed, Mohamed Ramadan, Hala Rady Ahmed, Israa M. S. Al-Kadmy, Yasmin N. Ramadan, Helal F. Hetta

**Affiliations:** 1 Department of Microbiology and Immunology, Faculty of Pharmacy, Minia University, Minia, 61519 Egypt; 2 Department of Microbiology and Immunology, Faculty of Pharmacy, Deraya University, Minia, 11566 Egypt; 3 Branch of Biotechnology, Department of Biology, College of Science, Mustansiriyah University, POX 10244, Baghdad, Iraq; 4 Department of Microbiology and Immunology, Faculty of Pharmacy, Assiut University, Assiut, 71515 Egypt; 5 Department of Medical Microbiology and Immunology, Faculty of Medicine, Assiut University, Assiut, 71515 Egypt

**Keywords:** SARS-CoV-2, Risk factors, Opportunistic pathogens, *Mucormycosis*, Control measures

## Abstract

The severe acute respiratory syndrome coronavirus-2 (SARS-CoV-2) pandemic is still difficult to be controlled. The spread of this virus and the emergence of new variants are considered a great challenge worldwide. Disturbance in infection control guidelines implementation, use of steroids, antibiotics, hospital crowdedness, and repeated use of oxygen masks during the management of critically ill COVID-19 patients lead to an increase in the rate of opportunistic infections. So, patients need to fight both the virus with its different variants and opportunistic pathogens including bacteria and fungi especially patients with diabetes mellitus, malignancy, or those who undergo hemodialysis and receive deferoxamine. During the pandemic, many cases of Mucormycosis associated with COVID-19 infection were observed in many countries. In this review, we discuss risk factors that increase the chance of infection by opportunistic pathogens, especially fungal pathogens, recent challenges, and control measures.

## Introduction

The latest coronavirus pandemic is caused by the severe acute respiratory syndrome coronavirus 2 (SARS-CoV-2). The upper respiratory tract is the primary site of COVID-19 infection and fluctuates in severity from infection that is asymptomatic or very minimally symptomatic to lower tract destruction and may complicate to mild to serious bilateral pneumonia (Farghly et al. 2022; Khalaf et al. 2022; Moubarak et al. 2021). According to the latest report from World Health Organization (WHO), more than 760 million cases and 6.9 million deaths have been documented worldwide since December 2019, although the true figure is estimated to be higher (WHO 2023). Individuals with serious illnesses may experience respiratory distress and respiratory failure, requiring hospitalization to the intensive care unit (ICU) and mechanical respiratory support (Ranieri et al. 2012; Khalaf et al. 2022; Abdelaal et al. 2023). The high rate of morbidity and mortality associated with long periods of ICU admission may be attributed mainly to the increased chance of secondary infections. These infections include mainly bloodstream infections, pneumonia, and to a lesser extent urinary tract infections and catheter-associated infections (McMullen et al. 2020; World Health Organization 2020).

**Fig. 1:**
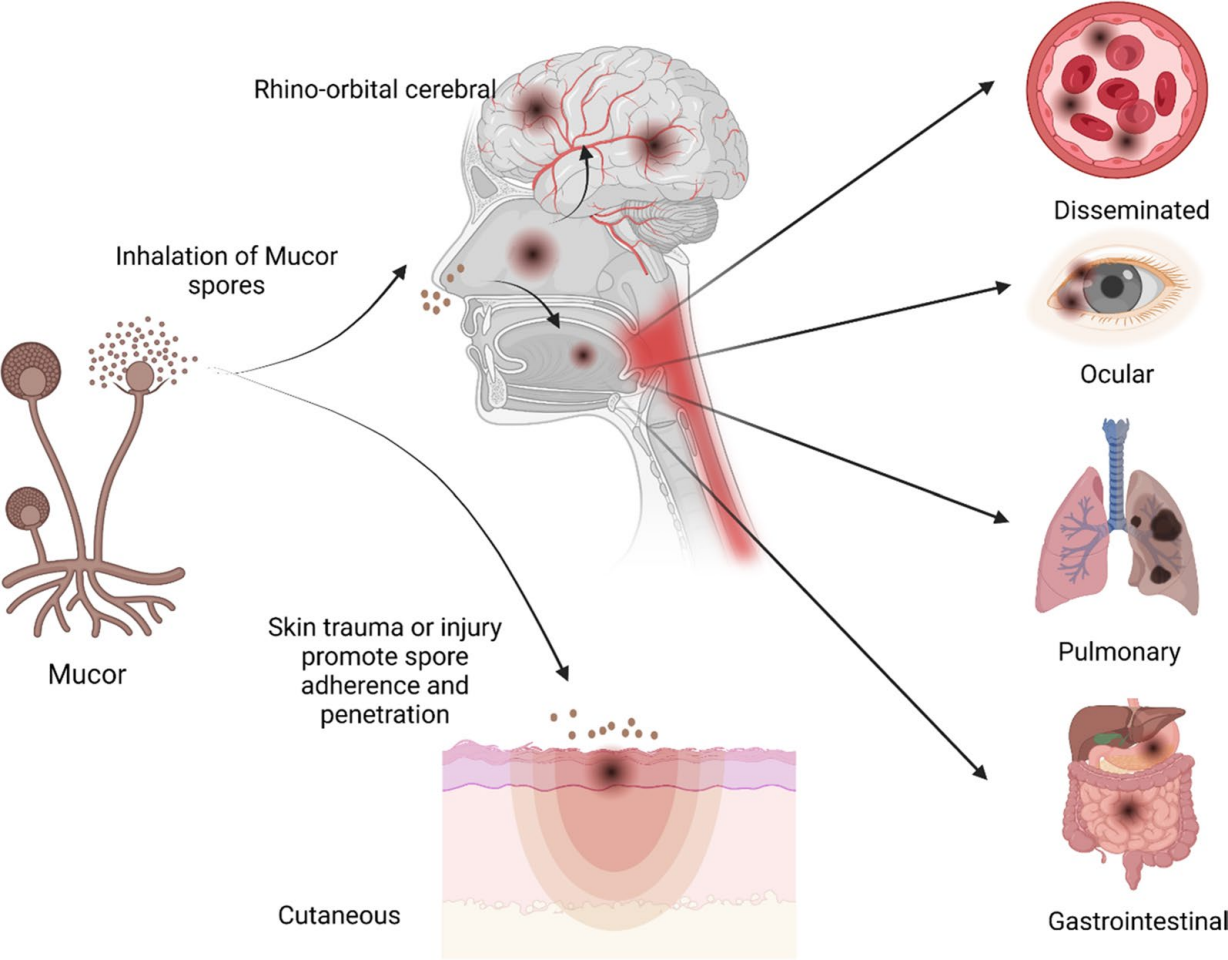
Ways for pathogen entry and common clinical presentation. Mucor can enter the body through spore inhalation from environment and direct contact of spores with skin burn, injury or trauma and cause cutaneous mucormycosis. After inhalation, spores germinate in sinus and spread to other sites and different organs causing different types of clinical manifestations as rhino-orbital cerebral, ocular, pulmonary, gastrointestinal or disseminated mucormycosis

Mucormycosis is a devastating opportunistic invasive fungal illness, often observed in patients with impaired immune systems. It is classified as filamentous mold from the zygomycete family (Reid et al. 2020). Mucormycosis ranks third in severity behind candidiasis and aspergillosis in the world. The progressive rise in mucormycosis cases over the past 20 years is mostly attributable to an elevation in the number of patients administering immunosuppressive drugs and antibiotics. Consequently, an extraordinary number of mucormycosis cases have emerged after the second COVID-19 outbreak. Mucormycosis can manifest clinically in several ways, including disseminated, pulmonary, gastrointestinal, cutaneous, renal, and rhino-orbito-cerebral (Fig. 1). There have also been reports of infections of the bones, heart, ear, parotid gland, uterus, urinary bladder, and lymph nodes. The most prevalent form of mucormycosis, rhino-orbito-cerebral mucormycosis, is most frequently seen in those with diabetes and those who have diabetic ketoacidosis, followed by the pulmonary form. It is thought that those who breathe in spores from the air experience sinus and lung congestion. This led to dysregulation in the immune reaction resulting from inflammation, cytokine storm, and microvascular clotting, which will exacerbate the presence of fungus infections (Prakash and Chakrabarti 2019; Skiada et al. 2020; Song et al. 2020).

Mucormycosis is commonly treated with invasive surgical procedures as well as antifungal medications such as amphotericin B, posaconazole, or isavuconazole. By promoting COVID-19 immunization, following recommended protocols when prescribing steroids for COVID-19 treatment, and monitoring blood sugar levels in obese and diabetic patients who have COVID-19, the risk of COVID-19-associated mucormycosis may be reduced (Beshbishy et al. 2020, 2021). Moreover, early recognition and management are critical to enhancing outcomes. Additionally, Patients with COVID-19 should be evaluated for the potential of mucormycosis even if they don't have the typical risk factors for the condition (Farghly et al. 2022).

### COVID-19 pandemic outcomes and risk factors enhancing the secondary microbial infection

ICU infection is defined as a new-onset infection that appears after more than 48 h of admission to the ICU for which a new antibiotic regimen is started. It is well known that the widespread and sustained transmission of SARS-COV-2 resulted in a high rate of hospitalization (COVIDSurg Collaborative 2021). It was found that a great increase in the total capacity of hospitals to provide the required needs for COVID-19 patient care, especially with the great need for mechanical ventilation support and oxygenation results in great pressure on the ICU. As a result of the increased capacity of ICUs, infection control measures were damaged and a reduction in the surveillance process for these measures was observed which give rise to other complications such as hospital-acquired infections with opportunistic pathogens (Baccolini et al. 2021; COVIDSurg and GlobalSurg 2022).

**Fig. 2:**
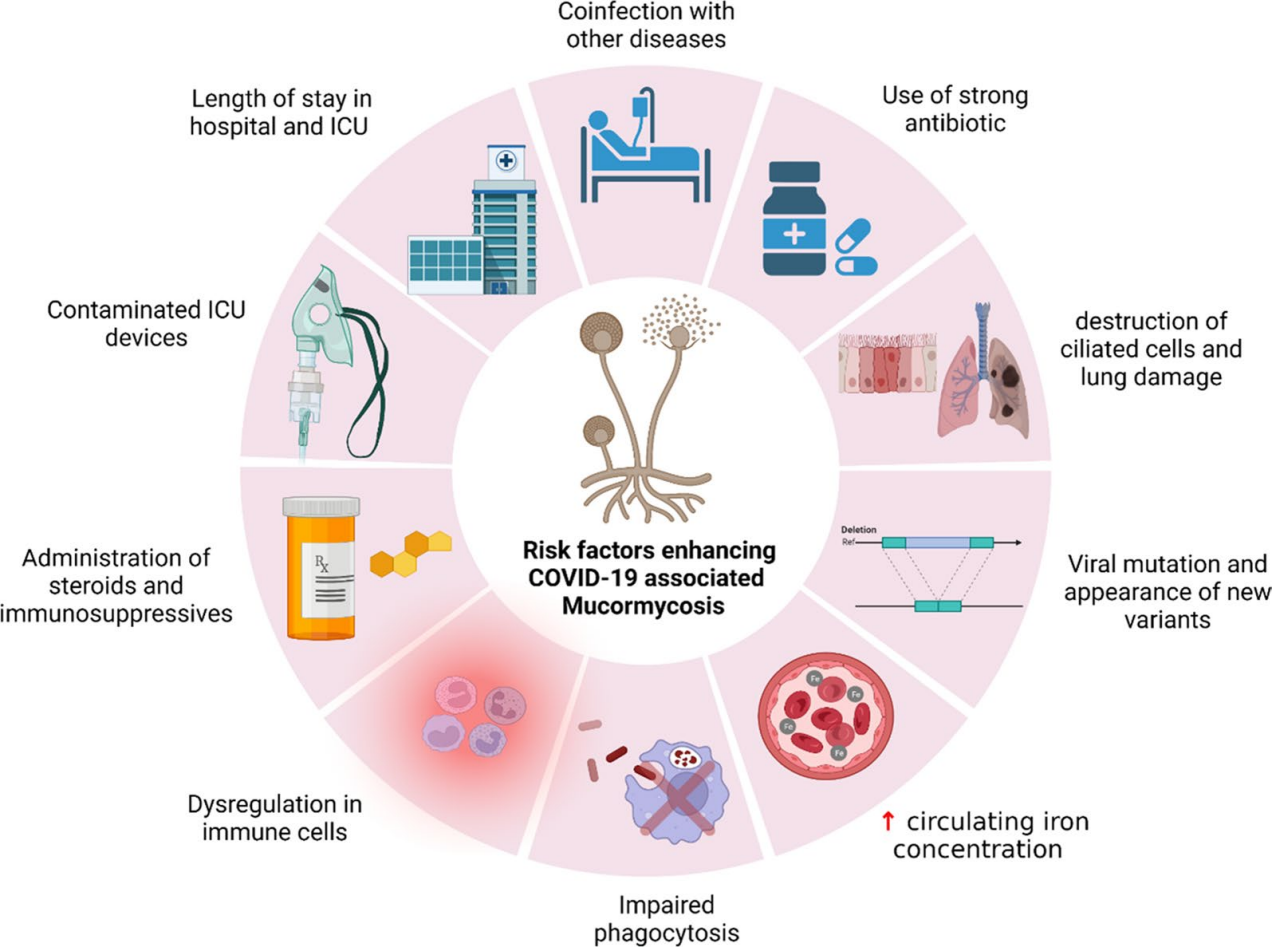
Risk factors enhancing COVID-19 associated mucormycosis. Many risk factors enhancing ICU COVID-19 associated mucormycosis as hospitalization and length of stay in ICU, contaminated oxygen mask and other ICU devices, taking strong antibiotics, steroids and immunosuppressives, destruction of ciliated cell in nose and lung damage, increase circulating free iron concentration, dysregulation in immune cells, impaired phagocytosis and viral mutation and appear of new variants

In addition, many factors may increase the probability of ICU-associated infections during the COVID-19 pandemic including the presence of underlying disease, the length of stay in the hospital, cross-contamination of microorganisms among patients as a result of the crowd, the number of medical staff not suitable for the high rate of COVID-19 infected patients in need to hospitalization, use of corticosteroids, the use of strong antibiotics that represent a great pressure for the selection of highly resistant microorganisms, and the administration of highly immunosuppressive drugs such as Janus kinase inhibitors or IL-6 receptor inhibitors to treat COVID-19 patients (McCabe et al. 2020; Musuuza et al. 2021; Abdellatif et al. 2021). In addition to the previous risk factors, superinfection, and co-infection are associated with poor outcomes showing a high mortality rate (Musuuza et al. 2021). Also, viral infection leads to the destruction of ciliated cells, acute inflammation, and damage to lung tissue which is followed by variable immune responses among patients resulting in the susceptibility of the respiratory cells to secondary infections (Medell et al. 2012; Hendaus et al. 2015) (Fig. 2).

Another challenge is the ability of viruses to mutate. Viruses constantly change through mutation and sometimes these mutations result in a new variant of the virus. The more possibilities a virus has to spread, the more possibilities it has to mutate (Moubarak et al. 2021; Dar et al. 2020). So, the implementation of infection control measures, vaccination, and isolation of cases can decrease the rate of mutants’ development. Some variants emerge and disappear while others persist. All variants of the COVID-19 virus globally were monitored by a public health organization and CDC. These variants were classified as variants being monitored, variants of interest, variants of concern, and variants of high consequence depending on their ability to spread easily, the severity of symptoms, response to treatments, and how vaccines can protect against these variants (Castonguay et al. 2021).

Regarding the previous risk factors and the probability of secondary infections, severe cases, and immunocompromised patients should be tested for fungal pathogens microscopically, serologically, histopathologically, or by PCR techniques followed by antifungal susceptibility testing (AST) to start antifungal therapy at earlier stages or using empirical antifungal therapy if AST is difficult to be performed (Song et al. 2020).

#### Mucorales and spread of Mucormycosis

*An outbreak is defined as an increase in the incidence of infection in a certain area in a short period. Due to the low incidence of Mucormycosis, the emergence of 2 or more cases in a short period is considered an outbreak (*Antoniadou 2009*).*

The order Mucorales is a group of ancient fungi classified in the subphylum Mucoromycotina (Nicolás et al. 2020). The Mucorales order consists of 260 species in 55 genera including 38 species that can cause human infections (Wijayawardene et al. 2018). Mucorales are opportunistic fungi that can enter the human body and progress to cause infection (de Hoog et al. 2018) Mucorales infection may be a hospital or community-acquired infection. Their infections originate from (1) contaminated medical devices such as bandages, wooden tongue depressors, and linen (2) inhalation of aerosolized spores of contaminated air via ventilation systems, construction works, or water damage (3) traumatic inoculation of soil or foreign bodies (4) contact with contaminated plant material. It should be considered that person-to-person transmission is uncommon but cannot be excluded because Mucorales can sporulate on wounds (Mitchell et al. 1996; Walther et al. 2020). Infection occurs due to the inhalation of fungal spores or the contamination of injured skin with spores. Mucormycosis infection does not spread from patient to patient or animal to human but can be transported with the aid of a contaminated environment. Many fungal species can cause Mucormycosis such as *Rhizopus* species, *Mucor* and *Rhizomucor* species, *Syncephalastrum* species, *Cunninghamella bertholletiae*, *Apophysomyces* species, and *Lichtheimia* (formerly *Absidia*) species. Other fungal strains that can cause secondary fungal infections are *Aspergillus* spp., *Penicillium* spp., and *Candida* spp. (Nambiar et al. 2021).

Mucorales are widely spread in nature (subphylum Mucoromycotina, Phylum Glomeromycota). They are characterized by aseptate hyaline hyphae, with wide branching angles and large diameters, and reproduce sexually by the formation of zygospores and asexually by non-motile sporangiospores (de Hoog et al. 2005). Although Mucormycosis has a low incidence that ranged from 0.005 to 1.7 per million, a significant increase in its incidence occurs in coronavirus pandemics (Zhang et al. 2003). During the 2003 SARS-CoV pandemic, fungal infections represented 14.8 to 27%. Moreover, it was found that the main cause of death associated with SARS infections since 2003 was due to co-infection with fungal pathogens with a mortality rate ranging from 25 to 73.7% (Li and Pan 2003; Song et al. 2020).

In a recent study, White et al. (2021) reported that out of 135 COVID-19 patients, 26.7% were infected by invasive fungal infections. Sahoo et al. (2021) reported that during the second wave of the COVID-19 pandemic, 59 cases of Mucormycosis were detected around the world. They found that 42.3% of cases were specific to India followed by the United States of America (16.9%), Iraq (8.4), Bangladesh (6.7%), Iran (6.7%), Paraguay (3.3%), and 1.6% each from Brazil, Mexico, Italy, UK, China, France, Uruguay, Turkey, and Austria. Farghly et al. (2022) reported that out of 433 patients admitted to Assuit University Hospital between March 2021 and July 2021 with definite covid-19 infection, 33 (7.63%) patients were infected with Mucormycosis. These patients suffered from diabetes mellitus (DM) or hypertension or were smokers. The high mortality rate associated with co-infection with fungal pathogens was attributed to the that the detection of fungal infection associated with COVID-19 infections is always not considered or delayed. During the recent pandemic, a high mortality rate was found among COVID-19 patients suffering from Mucormycosis. Testing for co-infection with bacteria or mycoplasma was the first to be considered but testing for fungal co-infection was delayed or neglected resulting in the starting of antibiotic therapy (Chaturvedi et al. 2018; Guan et al. 2020). Also, symptoms of Mucormycosis resemble those of other fungal infections resulting in misdiagnosis. Difficulty in the diagnosis and the need for tissue biopsies to be submitted to histopathological or microbial examination for the confirmation of infection (Baldin and Ibrahim 2017).

### The role of some underlying diseases conditions in increasing the chance of the development of Mucormycosis

**Fig. 3:**
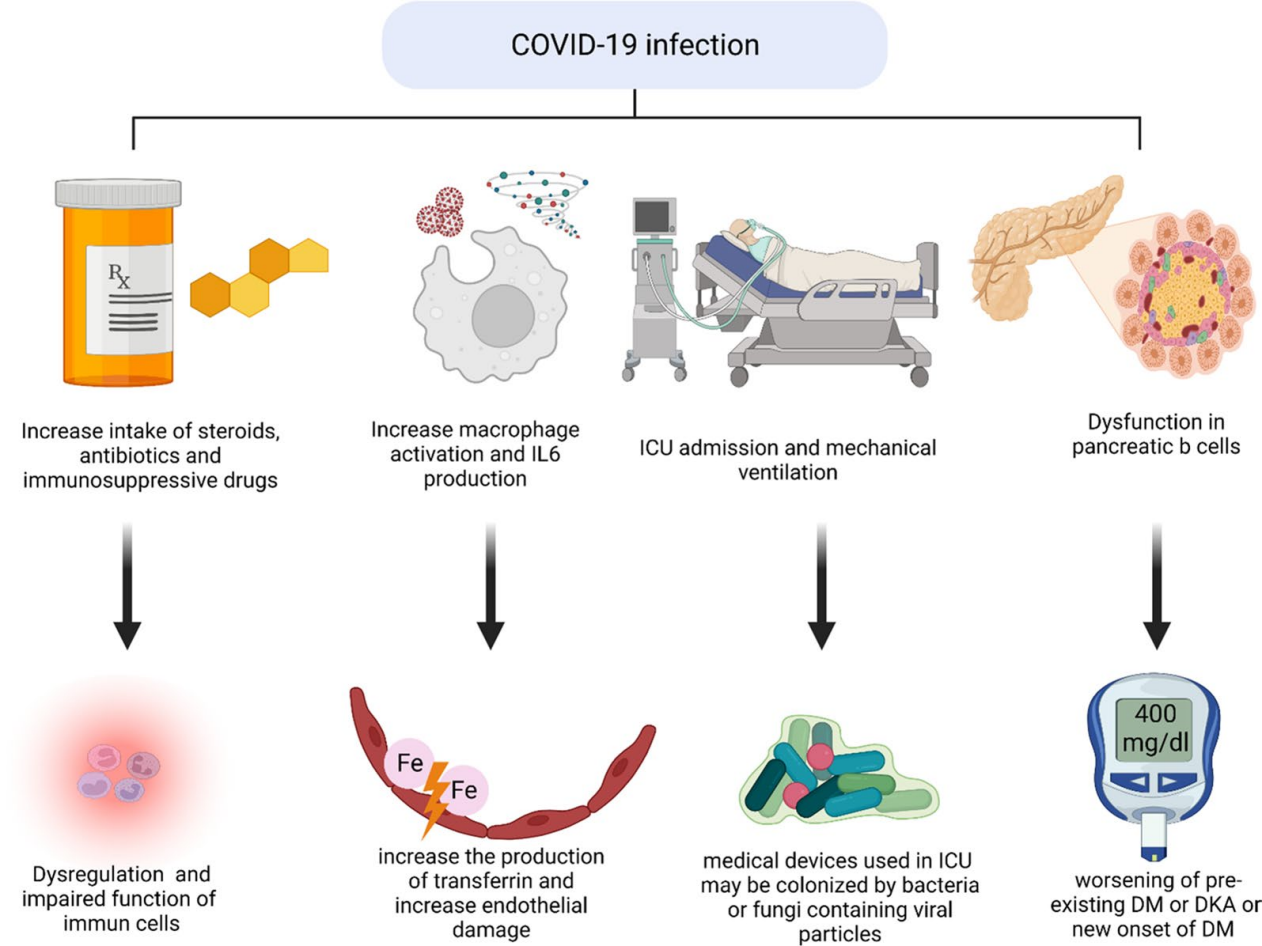
Effect of COVID-19 with co-existing disease in developing Mucormycosis. When COVID-19 co-existing with other disease, enhance development of 2ry infection as Mucormycosis through: (1) increase administration of steroids and other immunosuppressive drugs that decrease patient immunity through dysregulation of immune cells (2) increase in macrophage activation and cytokines production as IL6 that enhance the increase in free iron concentration which cause endothelial damage and promote fungal invasion (3) ICU admission and mechanical ventilation in sever case increase risk of cross contamination (4) dysfunction of pancreatic beta cells that leads to hyperglycemia and may combined by diabetic ketoacidosis

Although Mucormycosis is a rare fungal infection, it can infect people with some underlying diseases or those who have some medications that affect the immune system. People at risk are those who have cancer,DM, hemodialysis patients, stem cells or organ transplants, skin injury due to surgery or burns and long-term corticosteroid therapy. Co-existing disease or infection with COVID-19 infection my lead to a drop in patient immunity, a change in metabolic balance as an increased concentration of iron or a decrease in pH, or even administration of medication that enhances the development of Mucormycosis (Fig. 3).

#### Acidic pH

Low pH due to acidosis is a suitable medium for the growth and germination of mucor species. High glucose concentration and high iron concentration increased endothelial damage facilitating the invasion of fungus in tissues (Dogra et al. 2021).

#### High concentration of free Iron

A high concentration of iron is an important factor in increasing Mucormycosis pathogenicity as it enhances its growth and hyphal development. DM associated with ketoacidosis, corticosteroid therapy, and hemodialysis are predisposing factors for iron overload and elevated serum iron. As hyperglycemia results in the glycosylation of transferrin and ferritin, reducing iron binding and increasing the level of free iron. In the case of acidosis, low pH increases ferritin levels due to increased production and a decrease in iron transport (Singh et al. 2021). COVID-19 patients showed an increase in inflammatory cytokines such as interleukin-6 which can also increase the production of transferrin and decrease iron transport and so increase intracellular iron concentration. Excessive increase in iron concentration produces free radicals that damage the endothelium, promote endothelitis, and encourage fungus invasion (Vlahakos et al. 2021). Hemodialysis patients receiving deferoxamine (iron chelator) are at great risk of developing Mucormycosis as Mucorales can use this drug as xeno-siderophore by detaching iron from and using it (Alloush et al. 2022).

### Low phagocytic activity

The virus is suspected to damage the pancreatic islet cells resulting in new-onset DM and worsening of pre-existing DM or DKA. In addition, cytokine storm indirectly fuels this by the development of insulin resistance. Hyperglycemia with or without DKA impairs neutrophil functions. Steroids decrease the phagocytic activity of WBC, and affect bronchoalveolar macrophages, and phagocytic processes including migration, ingestion, and phagolysosome fusion. Infection with SAR-CoV 2 causes lymphopenia, thrombosis, decrease in CD4 and CD8 T-cell levels that predispose to secondary infections (Ghosh et al. 2022).

### Biofilm formation

Although viruses are obligate intracellular parasites, many studies suggested the presence of virion particles as a secondary colonizer in bacterial or fungal biofilms in reservoir hosts. Also, they addressed that viruses could perform biofilms that are embedded in an extracellular matrix formed by the host microenvironment or are parts of bacterial or fungal biofilms.

In studies done by Sanjuán and Thoulouze (2019), Thoulouze and Alcover (2011), and Von Borowski and Trentin (2021), authors observed the presence of extracellular viral particle assemblies and considered them as viral biofilms. They explain these aggregates of viral particles as a protective pattern. They defined viral biofilms as a protective form of virion community embedded in a matrix of highly glycosylated proteins. As the presence of virion particles in biofilm form was found to enhance persistence and the chance of dissemination and infection. They reported that viral particles can spread in groups called collective infectious cells as the extracellular state of the virus-infected cells shas a great role in virus dissemination to infect other cells and start replication, during this step, viral particles have a great challenge represented by the host immune response or the physicochemical characters in the host microenvironment (Sanjuán and Thoulouze 2019; Thoulouze and Alcover 2011; Von Borowski and Trentin 2021).

Based on microscopical images, Tozzi et al. (2020) observed that SARS-CoV-2 presents as collective arrangements of multiple SARS-CoV-2 particles on the surface of the cultured cells. They suggested that viral biofilm starts as single viral particles aggregates in spherical assemblies. Then, interconnected network-like branches and nodes develop.

In another study done by Thoulouze and Alcover (2011), authors suggested the high affinity of beta coronaviruses to bind carbohydrates that result in increasing their affinity to polysaccharides presented on the matrix of microbial biofilms and to be embedded as a secondary colonizer and presented as biofilm patterns.

According to the previous findings, the presence of coronavirus biofilms or as a secondary colonizer in other microbial biofilms explain the severity of symptoms of SARS-CoV-2 associated with other microbial co-infection. Also, the high mortality rate may be attributed to the presence of microbial biofilms in medical devices used in ICUs which may be colonized by bacteria and/or fungi containing viral particles that can infect many patients receiving mechanical ventilation. Invasive Mucormycosis can gain access to CNS through vessels or from paranasal sinuses. Also, Mucorales have proteolytic, lipolytic, and glycosidic enzymes as well as their metabolites like alkaloids or mycotoxins as agroclavine which have destructive effects (Solanki et al. 2022).

### ICU unit’s environment and the implemented infection control measures as the first line of protection against infection

The main problem is that the ICUs of most hospitals are not designed or equipped to deal with airborne viral infections such as COVID-19 but also it may cause harm for the medical staff (Ramakrishnan and Sharma 2020). First, the location of the ICU concerning COVID-19 patients should be away from other critical ICUs. Also, these units should have separate entry, exit, and specific lifts and stairs. ICU units should have areas that can be used as donning and doffing areas near the entrance of the ICU. It is important to save a place for showering after doffing and before leaving the ICU area. There should be a place for the healthcare team to rest to avoid burnout syndrome (Sharma et al. 2020).

ICUs should be designed to accept the increase in bed capacity by 20% (in case of a surge in patients). Hand washing using a no-touch sensor is important to avoid transmission of infection. Disposable linen should be placed in 5% sodium hypochlorite. Providing resting rooms and chambers for doffing by UV lamps to disinfect surfaces. The presence of means of communication to facilitate communication between patients and their families is essential for their support and to avoid the risk of a physical visit. Rooms should have fresh air exchange and central air conditioning should be avoided as it may result in the spreading of infection (Jog et al. 2020). An important issue is the choice of healthcare free from comorbidities and younger than 45 years of age as recommended by the CDC (2020). Nurses should be highly qualified, and the care team should include experts in infectious disease and respiratory therapists (CDC 2020).

### Hospital disinfection

The main cause of ICU-acquired infection is non-compliance with infection control measures. The necessity for the removal of the organic or non-organic matter before disinfection with the commitment of wearing protective personnel equipment (PPE), surfaces should be disinfected with 1% sodium hypochlorite or 70% ethyl alcohol. Well-trained staff should deal with dead bodies including those who work in crematoriums/burial grounds. Sample collection should be with well-trained staff (WHO 2020).

A great challenge that faces the health team workers is the decontamination and sterilizing of mechanical ventilators, anesthesia machines, and suction devices. The high-quality viral filter will protect the internal components of the anesthesia machine from contamination. Single-use items must be discarded and not used between patients. As the medical staff working in the ICU should be aware of the guidelines for using this equipment to avoid the accumulation of condensate inside the device or the drain of condensate toward patients. The internal parts should be disinfected by high-level liquid disinfectant or pasteurization by submerging these parts in water at 70֯C for 30 min followed by drying in a hot-air drying cabinet before storage. The presence of condensate makes the environment suitable for the growth of microbial communities containing bacteria and fungi (Bottiroli et al. 2021).

According to the previous findings, the emergence of Mucormycosis results mainly due to the non-compliance to infection control measures concerning critical and semi-critical items for patients. So, ICUs should have a team that is expertise in how to disinfect and sterilize surfaces and devices with a high ability to follow up on these procedures daily. In our country, low resources, cleaning with water and detergent which may not be followed by disinfection is considered an important predisposing factor that may result in the emergence of secondary infections associated with ICU admission (Munoz et al. 2001). On the other hand, the quality of air should be assured to protect high-risk patients from exposure to fungal spores. Because contaminated oxygen supplies, respiratory equipment, humidifier water, and reused face masks are considered important causes of COVID-19-associated Mucormycosis (CAM) outbreak. Also, transmissions of Mucorales in susceptible patients may occur through hospital linen, contaminated catheters, armrests, and tongue depressors. According to CDC (2017) recommendations, impermeable barriers should be available between any construction area (if present) and ICUs to avoid dust-containing spores, water leakage areas in hospitals should be fixed, and potted plants and flowers should be away from areas containing high-risk patients. Many studies recommended the use of disinfectants with antifungal activity to avoid the spread of fungal spores. portable high-efficiency particulate air (HEPA) filters can be used in corridors and rooms. Using copper-8-quinolinolate in decontamination procedures and the use of trisodium phosphate detergent in the removal of dirt followed by a 10% bleach wipe for all vertical and horizontal surfaces (Streifel 2004). The use of benzalkonium chloride (quaternary ammonium compounds) for disinfection was recommended by many studies due to its high activity against all fungi in comparison to other disinfectants such as chlorhexidine and sodium hypochlorite. Also, Chlorine-based compounds showed activity against mold fungi while peracetic acid and alcohols showed activity against yeast form (Nowrozi et al. 2013; Roshan et al. 2021; Korukluoglu et al. 2006).

### Reported Mucormycosis worldwide

The latest systematic review and meta-analysis by Özbek et al. (2023) reported that corticosteroids (78.5%) and diabetes (77.9%) were the most common risk factors between cases and most reported cases for low- and middle-income countries. The most reported cases were from India (57%). Egypt (6%) comes in third rank after India and Iran. In another systemic review done by Singh et al. (2021), it was found that 80% of cases of Mucormycosis are associated with uncontrolled diabetes with or without ketoacidosis. Sen et al. (2021) showed that uncontrolled DM and the use of corticosteroids are the main risk factors for developing Mucormycosis in a study performed on 2826 COVID-19 patients associated with rhino-orbital-cerebral Mucormycosis (ROCM) from 120 treatment centers and union territories in India. They showed that it is difficult to predict if patients developing ROCM have a greater requirement of oxygen support as compared to those patients who do not develop ROCM. Avatef et al. (2021) reported that DM and corticosteroids are the main predisposing factors for COVID--19-associated ROCM among 12 patients identified from 12 October to 18 November 2020 in Iran. They suggested also that climate conditions especially in Autumn is associated with a high concentration of fungal spores in air compared to summer condition. Overall, India reported the majority of Mucormycosis cases linked to COVID-19, with at least 14,872 cases until May 2021 (Rudramurthy et al. 2021; Chao et al. 2022).

On the other side, no case of ROCM was reported in a large study of 5428 hospitalized patients with COVID-19 between March 2020 to May 2021, of whom 1027 were in the ICU (915 of 1027 received corticosteroids and 417 had DM) which was explained by the application of low dose corticosteroid protocol and strict glycemic control in addition to the quality of air in patients’ room (Mulakavalupil et al. 2021).

In addition to the previous findings, Healthcare-associated Mucormycosis was reported in many studies. Rammaert et al. (2012) reported 17 cases of Mucormycosis associated with contaminated Elastoplast in patients suffering from cancer, DM, and undergoing surgery. Christiaens et al. (2005) reported 5 cases of Mucormycosis associated with contaminated Elastoplast in the burn unit. Moreover, Mitchell et al. (1996) reported 4 cases of premature infants with Mucormycosis that resulted from contaminated wooden tongue depressors. Garner and Machin (2008) reported 2 cases of patients with leukemia due to water circuitry damage in their rooms. In a multi-center study performed in India to evaluate the effect of hospital environment on Mucormycosis infection, it was found that 21.2% of indoor air samples and 51.8% of outdoor air samples of hospitals were positive for Mucorales. They reported also that air samples obtained from individualized air-conditioning (AC) were more contaminated with Mucorales than air samples obtained from central AC with microfilters. A minimal spore count was observed in HEPA-filtered room air. Also, they showed that no Mucorales were isolated from hospital equipment or surfaces while 1.7% of Patients’ masks were positive for Mucorales and 8.1% positive for other fungi (Biswal et al. 2022).

In a report of a Mucormycosis outbreak, dust accumulation was observed on the return air grills in some rooms which tested positive for *Mucor* spp. In one patient’s room Mucor spp. isolated from air exhaust vent diffuser. Samples collected from air, toilet tiles, oxygen sockets and food trolleys, revealed growth of *Mucor* spp., which indicated dust contamination, poor disinfection measures and defective air filtration that is below standards for such a protective environment (Saleem et al. 2021).

Two hospitals, one in Belgium and one in Pakistan, had outbreaks of *Mucor* spp. from non-sterile bandages. Both hospitals subsequently recommended using only sterile bandages for deep, traumatic, and extensive wounds (Christiaens et al. 2005; Shakoor et al. 2011). In the Belgian hospital, 7 patients were found to be colonized with *Mucor* spp. Of those seven patients, five developed infections and three died. A third hospital in France used real-time PCR to test non-sterile bandages, detecting *Mucor* spp. (FrÚalle et al. 2018). In addition, Four cutaneous infections among infants in a neonatal ICU were also linked to wooden tongue depressors used as splints for intravenous and arterial cannulation sites (Mitchell et al. 1996).

Medical devices that have not been sufficiently sterilized may be contaminated with spores. One hospital ended an outbreak of postoperative bone Mucormycosis among three patients after improved sterilization of screws used in ligament reconstruction (Chaves et al. 2016). A hospital was investigating four cases of mycotic endocarditis, including one with *Mucor* sp. Isolated *Mucor* sp. Found in dust from an air conditioner duct and in air samples. The team found that the air filter used was likely insufficient to trap spores (Mishra et al. 1992). Another hospital reported Mucormycosis infections in premature infants in a neonatal ICU (NICU) adjacent to hospital construction, with inadequate barriers that allowed a higher density of mold spores measured in the unit than in a comparison area without construction (Krasinski et al. 1985). An outbreak investigation in a pediatric oncology ward in the UK reported by Garner et al., 2008, indicated that a defect in wall plastering was found in one of the patient’s toilets. Two children who developed Mucormycosis infections were in beds closest to a linen closet that shared a wall with a leaking shower. Testing by both air sampling and settling plates showed a high concentration of mucoromycetes spores near the water-damaged wall. The spore counts dropped to zero a week after remediation of the leak and wall with mold (Garner and Machin 2008). Several studies showed that Mucor species were detected in indoor and outdoor environmental air samples in Saudi Arabia (Kontoyiannis and Lewis 2011; Skiada et al. 2013). During 1976–2014, 28 published reports linked hospital construction to fungal disease outbreaks. Many found contamination of airflow systems and inadequate barriers at construction sites (Weber et al. 1990; Kanamori et al. 2015).

As shown before, the emergence of Mucormycosis is multi-factorial depending on the presence of underlying diseases, corticosteroids therapy, hospital environment and spore count in air, implementation of infection control measures, contaminated medical devices and climate conditions.

### Current challenges

The need for early treatment to increase the survival rate and the need to develop rapid and non-invasive protocols for the diagnosis of Mucormycosis, control of the progression of the disease, and the difficulty in intubation. The necessity for the development of new pharmaceutical dosage forms of antifungals such as liposomes, nanoparticles, or aerosols to decrease the need for surgical procedures to remove the infected tissues due to the poor bioavailability of antifungals to penetrate tissues and to decrease toxicity attributed to the long period and high doses of antifungal therapy that can cause renal impairment and the ability of some antifungal dosage forms to cause embolism after intravenous administration and ischemia of surrounding tissues (Abd et al. 2020; Mahmood et al. 2020; Dogra et al. 2021; Farghly et al. 2022).

In Conclusion, Mucormycosis or secondary fungal infections can be controlled by the implementation of infection control measures during COVID-19 pandemics, the introduction of enough well-trained infection control teams in hospitals, sterilization or high-level disinfection procedures for medical devices used in ICUs, proper choice of disinfectants with potent antifungal activity for the disinfection of surfaces and floors and the use of portable air HEPA filters in rooms of high-risk patients and corridors, isolation of cases, vaccination and the development of new strategies for therapy. In addition, it is necessary for severely ill patients or immunocompromised patients to be tested for fungal pathogens.

## Data Availability

Not applicable.
